# Some Observations upon Hereditary Tendencies and Transmissions

**Published:** 1881-10

**Authors:** Roger Marvin Griswold

**Affiliations:** Member and Fellow of the Connecticut State Medical Society, and of the American Medical Association, North Manchester, Ct.


### Independent Practitioner.

Vol. II.——*October*, 1881.——No. 10.

ARTICLE I.

### SOME OBSERVATIONS UPON HEREDITARY TENDENCIES AND TRANSMISSIONS.

By ROGER MARVIN GRISWOLD, M. D.,
Member and Fellow of the Connecticut State Medical Society, and of the American Medical Association, North Manchester, Ct.

[Continued from page 522.]

But we proceed to the next phase of constitutional transmissions, viz., the inherited mental qualities, gifts, capacities, aptitudes, idiosyncrasies, whims of the human mind, the psycological side of the subject. So obviously apparent is the fact that the mental aptitudes of the parents are transmitted to the children, that nearly every day we hear it said of some one, "He is just like his father, or his mother," or "He is a chip of the old block." So like sometimes are father and son, that we are justified in repeating the words of Burke on the younger Pitt, "He is not a chip of the old block, but the old block itself."

We observe instances in which peculiar traits of character, oddities of thought, moral obliquities and virtues as well, crop out in descendents even to several removes from the starting point. That which we call the mechanical talent often seems handed down from generation to generation, as also the mathematical talent. The talent for music, the musical ear—is particularly noticeable in some families.

We observe that some families are mostly all slow and deliberate, their thoughts and expressions are methodical, careful, easy, reticent in conversation, plodding in their mental labors, though none the less intellectual perhaps, and possibly more sound and reliable in their conclusions, than some others who are quick, witty, in some instances flighty, perhaps full of oddities, brilliant, with the mind always active in its workings. The subject of the hereditary transmissions of intellectual powers has been very ably discussed in a work by Francis Galton, published by the Appletons some ten or twelve years ago.

The fact that mental or moral qualities should be transmittable, may at first seem more unexplainable than that merely physical qualities should be so. That they *are hereditary we know*, and in point of fact they are no more unexplainable. The truth is, on all sides of the question under consideration, we are constantly coming in contact with things we do not comprehend nor understand. But before going further on this point, allow me to merely call your attention to the fact of hereditary insanity, or rather the predisposition to it, so very common as to have been observed in all ages, and among every civilized nation. After eliminating all cases of insanity proceeding from recognizable disease, and also all cases which can be traced to metal trouble or brain shock resulting from accidents, and all cases the outgrowth of indulgence in some vicious habits, the records of insane asylums, and observations of private practitioners assure us, that there still remains a large percentage of cases the result of direct hereditary transmission. For a number of generations it has been observed that certain families have this dread disease for a companion, while in other families it is seldom or never seen, or when seen is an accidental result, traceable to some particular cause and touching only the isolated individual.

But time will not permit us to extend our remarks upon this point, and we now come to the consideration of the question. "How is disease, or a tendency to disease, or how are hereditary tendencies, of any kind, transmitted? In what manner? By what law? How?

Although there are great lessons to be learned from the matter under consideration, lessons which involve the welfare not only of the present generation, but of the countless thousands who come after us, yet it is not my object to "point a moral, or adorn a tale" by spreading them out before you.

There are many things in the economy of the human system, both mental and physical, to the bottom of which we have not yet reached, and this is one of them, for when we ask "How is disease, or a tendency to disease, transmitted," we find we cannot answer, for we do not know.

But, as this question involves a question of animate existence, and the ills to which animate existence is heir to, it has naturally engaged much thought, evoked much experimentation, and evolved many curious theories In inquiring into a matter of this magnitude, we should remember that the majority of the theories advanced can be but theories only, and capable of no demonstrable proof. We must remember that we are but children in the narrow valley, who, looking out on the hill sides about us, imagine that we have seen the whole world. But when we essay to climb those sides, expecting soon to reach the edge, we find *mountains* following hills, peak after peak, till reaching the last *attainable* height, we gaze upon unending space. So, also, we in the valley of our first attainments, seeming to see all, have only to begin to *climb*, and we shall find eminence after eminence, till, reaching the zenith of human acquirement, we look out upon immeasurable and unending expanses beyond. And then. and then only, do we arrive at a comprehension of the *profundity of our ignorance.* Then and then only, do we begin to realize that there are fields so vast that we cannot reach out to them, heights so sublime that we can never explore their tops, and depths so deep, that the plummet of no human intellect can fathom them. And then, in humiliation, though not in despair, do we attain to some intelligent appreciation of the omnipotent power of the great architect of the universe, and thus appreciating, no longer wonder, that under the workings of his unchangable laws, operations are carried on, even beneath our very eyes, the processes of which we are not able to determine.

It is claimed by some that the term "tendency to disease" is not rightly used, but that what parents transmit to their children is not a "tendency to disease," but *the disease itself*, communicated by a disease germ. Now if this view were confined to the transmission of a few specific troubles, it might possibly hold good, but in order to sustain itself, it must show that not only in every case of scrofula, consumption, gout, &c., but also in every case of *every other*

hereditary disease or taint, it is true, and that it is true also in every *physiological transmission.* The acceptance of this view presupposed that there is a germ for every mental and moral quality, a deformity germ, a six fingered germ, a mathematical and a musical germ, an honesty and a dishonesty germ, a comical, a witty, and a melancholy germ, a cross-eyed germ, and an insanity germ. A little reflection will show us the absurdity of this view.

Let us here consider what is meant by the word "germ."

A germ is a "*first principle*," a starting point, from which anything springs. It is a tangible thing, although perhaps the smallest thing in God's creation endowed with life. It is not a power or force, as is meant when we speak of "vital force," which is intangible and incomprehensible, but it is a thing of life itself, from which another and a higher life may spring.

I have said that the smallest of God's creatures, endowed with vitality, was built up from a cell, not recognizably different from that from which man springs, and to illustrate let us call to our aid a little chemical assistance. Everything in nature is primarily composed of four elementary compounds, viz: Carbon, Hydrogen, Nitrogen, Oxygen.

The composition of strychnia, a most active poison and theine, the active principle of the tea plant, are the same, so far as possessing the same elementary compounds are concerned. They are both composed of C. H. N. O. So we see that nature, in her great laboratory, makes out of these four elementary bodies, a great variety of differing compounds, but how? In this simple way, viz: When she wishes to make strychnia she takes C.44, H.22, N.2, O.4, and when she wishes to make theine she takes C.8, H.5, N.2, O.2, and when we consider that the addition or subtraction of a single equivalent of any of these elementary compounds alters the character of the substance into whose composition they enter, and how almost absolutely endless are the changes that can be made by the transpositions of the equivalents, more numerous than the atoms of the material world, we begin to comprehend how it is that nature presents us with such countless variety from so few materials.

The chemist, who sometimes pushes his materialistic views to the extreme, and essays to explain all the mysteries of nature, sometimes denies the existence of a distinctive vital force, and observing that the living form is composed of chemical materials, assumes that it is endowed with life by some fortuitous combination of chemical equivalents.

*But no known arrangement of material agencies can impart life. No animate thing can spring from an inanimate existence.*

The principle of life is a special endowment, a mystery unfathomed and forever to be unfathomed by human intellect, one of the many mysteries never to be disclosed beyond the councils of the Infinite.

Our first grasp of the existence of vitality is in the germ cell of a living organism, either animal or vegetable. This cell, not visible to the naked eye, is a minute mass of colorless liquid, surrounded by its own cell wall. By a mysterious power with which it is endowed, it is built up by the addition of other and still other cells to itself. Chemically speaking, this cell is composed of the same four elementary bodies of which we have been speaking, and between the cell which grows up by the absorption of other materials from without into a sturdy oak, and the cell which grows up into a summer weed, there is no visible difference. We cannot predicate of one cell that it will develope into a nettle and of another that it will develope into an apple-tree, neither can we predicate of a third that it will eventuate into a man, and of a fourth that it will become a tad-pole, for between the cell that developes into a man and the cell that perpetuates the race of tad-poles the most powerful microscope has failed to detect any difference, and chemistry has discovered none.

They are all C. H. N. O.

Yet, out of these, in the progress of development, we are brought face to face with wonderful differences, and thus we are brought to the point of asking:

May not the change consist simply in the interchange of the equivalents, or chemical compounds of the first germ cell? We have seen how, in the vegetable world, nature produces such diverse compounds as theine and strychnia from the same materials, and may we not, following the same diversity of equivalents, secure not only all the different orders that exist in animate things, but also all the shades of difference that exist in different specimens of the same species?

And not only this, but instead of having a special germ peculiar to each specific transmittable disease, why is it not reasonable to suppose that it is accomplished by an undetectable difference in the first germ cell, or in all the germ cells of the body ? (This is merely a theory and speculation, but one which we have never seen advanced before ; we can therefore claim for it originality, if nothing else.)

It may serve to show the difficulties that surround the question of "How are disposition, genius, special and mental aptitudes handed down from generation to generation ? How are peculiarities of physique and temperament transmitted ?" It may show the difficulty of determining how consumption, scrofula, insanity, gout, &c. are continued from family to family. It may show that the public expect too much of the medical profession when they ask a satisfactory explanation of all these phenomena surrounding our existence, and that the physician or scientist who assumes to answer all the many questions as to the why and wherefore of many things, is presuming upon the ignorance of others.

We can only reply, in regard to certain conditions and things, that they are so, that *why* and *how* they are so we do not know, and all else regarding them is but theory, advanced as rational one day, to be completely upset by some new theory the next.

